# Cognitive Adaptation to Stress and Trauma: The Role of Self-Appraised Problem-Solving in Posttraumatic Stress Disorder

**DOI:** 10.1177/24705470231189980

**Published:** 2023-07-25

**Authors:** Anita Padmanabhanunni, Tyrone B. Pretorius

**Affiliations:** Department of Psychology, 56390 University of the Western Cape, Cape Town, South Africa

**Keywords:** mediation, problem-solving appraisal, perceived stress, posttraumatic stress disorder, COVID-19, South Africa

## Abstract

**Background:**

Cognitive appraisals play a fundamental role in mental health outcomes following exposure to trauma. Appraisals influence emotional reactions, coping responses, and adaptation to stress and represent a modifiable factor that can serve as a central focus for intervention. Most studies have primarily focused on the role of dysfunctional cognitions in the persistence of posttraumatic stress disorder (PTSD). In this study, we extend research in this area by investigating the role of problem-solving appraisal, an adaptive cognitive strategy, in the association between stress and PTSD.

**Methods:**

A total of 322 participants completed the Perceived Stress Scale, the problem-solving inventory (PSI), and the PTSD Checklist for DSM-5. Descriptive statistics were generated, and intercorrelations and mediation analysis were performed.

**Results:**

Problem-solving confidence and personal control partially mediated the relationship between stress and PTSD. However, contrary to existing research, the approach-avoidance style, which is a subscale of the PSI, did not mediate the relationship between these variables.

**Conclusion:**

Interventions for PTSD should incorporate a complementary focus on developing and increasing adaptive cognitions pertaining to personal control and confidence in problem-solving abilities. This could potentially form part of a broader process of rebuilding the individual's cognitive worldview following exposure to trauma.

## Introduction

Empirical evidence compellingly suggests that cognitive appraisals play a pivotal role in mental health outcomes. Assessing and evaluating life events have significant implications for emotional responses, coping behaviors, and positive or negative adaptation to adverse events.^1^ According to the seminal theory of stress and coping of Lazarus and Folkman,^1^ cognitive appraisals in stress responses are divided into primary appraisals, which entail an immediate assessment of whether the stressor threatens one's well-being or goals, and secondary appraisals, which entail an evaluation of the individual's ability to cope with the stressor and the internal (e.g., sense of coherence) and external (e.g., financial resources and social support) resources available to them. Most research on cognitive appraisals has focused on the role of dysfunctional cognitions in the onset and maintenance of mental health disorders.^2,3^ For example, consistent findings have indicated that appraisals of loss of a vital investment (e.g., a romantic relationship or job opportunity) and of the self as being deficient and unworthy underlie depression,^4^ whereas dysfunctional appraisals regarding one's ability to cope with or personally influence negative events are associated with the persistence of generalized anxiety disorder.^5^ According to studies on suicidality,^6,7^ negative subjective appraisals of social problem-solving ability and negative perceptions of the availability of social support predict suicidal ideation. In posttraumatic stress disorder (PTSD), significant differences in appraisals have been found to account for the persistence of the disorder following exposure to traumatic events.^8^ According to cross-sectional and prospective longitudinal studies of PTSD^9,10^ following exposure to different types of traumatic events, problematic appraisals of trauma and its sequelae, as well as perceptions of ongoing threat, perpetuate post-trauma symptomology. Negative appraisals of personal vulnerability and future harm have also been found to account for unique variances in PTSD.^11^

Although many studies have explored negative appraisals, only a few studies have investigated the protective role of adaptive appraisals in mental health outcomes. Adaptive appraisals are not simply the absence of maladaptive cognitions but are rather specific thoughts and beliefs that positively influence outcomes.^3^ According to the seminal study of Beck et al.^12^ on cognitive therapy, psychological distress is determined by the interplay between negative appraisals of adverse events and positive appraisals of personal control (PC) and coping ability. The presence of positive or adaptive cognitions can help individuals tolerate negative appraisals or re-appraise situations in a way that facilitates coping. For example, benevolent religious re-appraisals of stressful circumstances, as a valuable learning opportunity, have been found to aid coping among young adults [8]. Empirical support for the role of adaptive appraisals has largely been derived from research on self-efficacy, which is defined as the belief that one can effectively cope with adverse events. Shahrour and Dardas^13^ reported that self-efficacy significantly predicted psychological distress and acute stress disorder among nurses during the COVID-19 pandemic. Self-efficacy has also been reported to partially mediate the relationship between cyber-victimization and depression.^14^ Luberto et al.^15^ examined the relationship between mindfulness and emotional dysregulation and found that low coping self-efficacy predicted high emotional dysregulation among adults.

In this study, we investigated the role of self-appraised problem-solving ability in the relationship between stress and PTSD in a sample of South African university students. Self-appraised problem-solving ability refers to an individual's appraisal of their own ability to solve problems and effectively respond to stressors, and it entails a secondary appraisal process.^16^ The majority of studies on the construct of problem-solving have focused on problem-solving ability, which refers to an individual's capacity to effectively and efficiently use internal and external resources to cope with stressors. Problem-solving ability entails both cognitive and behavioral processes including understanding the nature of the problem, generating and assessing possible solutions to the problem, evaluating the resources available to cope, and implementing strategies and assessing their effectiveness in managing the problem. Furthermore, existing studies^17,18^ have predominantly investigated the association between problem-solving ability and mental health or psychological adjustment, since deficits in this area confer a risk for depression, suicidality, and physical health problems. Comparatively fewer studies have investigated the role of self-appraised problem-solving ability in mental health outcomes. Furthermore, a significant portion of studies on problem-solving appraisal has been conducted in the late 20th century (i.e., 1970-1999), and there is a need for additional research in this area. For example, Elliott et al.^19^ studied psychological adjustment following spinal cord injury and discovered that higher self-appraised problem-solving ability was associated with lower levels of depressive behavior and psychosocial impairment. Kerns et al.^20^ investigated patients with chronic pain and reported that decreased self-appraised problem-solving ability was associated with increased pain, depression, and disability. In a study exploring the association between problem-solving skills and suicidality by examining the role of problem-solving appraisal, Dixon et al.^21^ discovered that reduced problem-solving appraisal independently predicted suicidal ideation and hopelessness.

PTSD develops in response to exposure to traumatic events and is characterized by symptoms of intrusive re-experiencing (e.g., flashbacks and nightmares of the trauma), physiological hyper-arousal, changes in cognition and mood, and cognitive and behavioral avoidance strategies.^8^ Evidence suggests that university students are at an increased risk of experiencing traumatic events, with exposure rates ranging from 48% to 95%, thus placing them at a risk of developing PTSD.^22,23^ In South Africa, McGowan and Kagee^24^ investigated the prevalence of 14 potentially traumatic events among university students and discovered that approximately 90% of the sample reported exposure to 1 or more traumatic events in their lifetime. In a more recent study, Padmanabhanunni^25^ reported a prevalence rate of 97.6% for trauma exposure among a sample of students. They reported that physical assault, transportation accidents, and sexual assault were the most common types of trauma. Bryant^26^ reported that, despite the high prevalence of exposure to trauma, a significant proportion of the population did not develop PTSD. These findings highlight the potential role of adaptive appraisals that may confer protection by buffering the impact of dysfunctional cognitions or facilitating their re-appraisal. Furthermore, a core emotional response implicated in the persistence of PTSD is helplessness and powerlessness, which are evoked by appraisals of not being able to respond effectively (i.e., problem-solve) in the context of the traumatic event.^27^ Hence, appraisals of problem-solving ability may be a central component in the persistence of the disorder and have implications for intervention. In this study, we hypothesize that the dimensions of problem-solving appraisal (problem-solving confidence (PSC), PC, and approach-avoidance style (AAS)—see “Instruments for descriptions”) would mediate the relationship between perceived stress and PTSD.

## Methods

### Participants and Procedure

The study sample consisted of 322 university students who chose to respond to an invitation that was sent to a randomly selected sample of 1500 students from a higher educational institution located in the Western Cape in South Africa. The response rate was 21.5%, which resulted in a margin of error of 5.31% (95% confidence interval [CI]). An electronic survey was conducted with the instruments described below using Google Forms and distributed to the participants for completion online. The majority of the sample were women (77%) who lived in an urban area (87.3%). The mean age of the sample was 26.01 years (SD  =  10.19). Most of the participants were vaccinated against COVID-19 (86.6%), and 40.7% had experienced the loss of a family member due to COVID-19.

### Instruments

The electronic survey included the perceived stress scale (PSS),^28^ the problem-solving inventory (PSI),^29^ and the PTSD Checklist for DSM-5 (PCL-5).^30^ Given the length of the survey, only a brief demographic questionnaire was included.

The PSS is a 10-item measure of perceived stress that uses a 5-point scale ranging from 0 (*Never*) to 4 (*Very often*). A sample item is “In the last month, how often have you felt nervous and stressed?” Cohen^28^ reported satisfactory reliability estimates (α  =  0.84–0.86) in 3 different samples and provided evidence of validity through correlations between perceived stress and life-event scores and depressive and physical symptomatology, among other factors.

The PSI is a 32-item measure of participants’ appraisal of themselves as effective or ineffective problem-solvers. It uses a 6-point scale ranging from 1 (*Strongly agree*) to 6 (*Strongly disagree*). The PSI consists of 3 subscales: PSC, AAS, and PC. PSC refers to a participant's confidence in their problem-solving abilities (e.g., “I have the ability to solve most problems, even though initially no solution is immediately apparent”). AAS describes the general disposition to approach or avoid problem-solving tasks (e.g., “After I solve a problem, I do not analyze what went right and what went wrong”). PC describes a participant's belief in their control over their own behaviors and emotions while solving problems (e.g., “Sometimes I do not stop and take time to deal with my problems but just kind of muddle ahead”). In the PSI, high scores reflect self-appraised ineffective problem-solving. Heppner^29^ reported an internal consistency coefficient of 0.90 for the total scale, with subscale reliabilities ranging between 0.72 and 0.85. Validity was also established through the relationship between problem-solving appraisal and a participant's rating of their problem-solving skills, as well as the ability of the PSI to differentiate between participants who had received training in problem-solving skills and those who had not. In South Africa, the reliability coefficients for the PSI range between 0.83 and 0.89.^31–33^

The PCL-5 is a 20-item assessment tool used to evaluate the severity and presence of PTSD symptoms. It is scored on a 5-point scale that ranges from 0 (*not at all*) to 4 (*extremely*). A sample item from the PCL-5 is “How much have you been bothered by repeated, disturbing dreams of the stressful experience?” Blevins et al.^30^ reported estimates of internal consistency of 0.94 and 0.95 for 2 distinct samples of college students. They also provided evidence of validity through correlations between the PCL-5 and other measures of PTSD and depression. In South Africa, Padmanabhanunni and Wiid^34^ reported an estimate of internal consistency of 0.93 for the PCL-5.

### Ethics

This study was conducted in accordance with the guidelines outlined by the Declaration of Helsinki and was approved by the Humanities and Social Sciences Ethics Committee of the University of the Western Cape (ethics reference number: HS22/2/9, February 2022). Participation was voluntary, and no incentives were offered for participation. The participants completed the survey anonymously and provided informed consent on the landing page of the survey link.

### Data Analysis

All statistical analyses were performed using IBM SPSS Statistics version 28 for Windows (IBM Corp., Armonk, NY, USA). Descriptive statistics (means and standard deviations), intercorrelations between study variables (Pearson's *r*), and estimates of internal consistency (α and ω) were examined, and a mediation analysis was conducted. Coefficient ω was obtained using the OMEGA macro in SPSS.^35^ A mediation analysis was conducted using the PROCESS macro written by Hayes for SPSS.^36^ The significance of the indirect effects of perceived stress on PTSD was evaluated using bootstrapped 95% CIs. The results indicated that gender and age were associated with perceived stress, problem-solving appraisal, and PTSD (as reported in the “Results” section). Therefore, they were included as covariates in the mediation analysis. A post-hoc power analysis specifying a medium effect size and α  =  0.05 indicated that the achieved power was 0.99.

## Results

Table 1 presents the descriptive statistics, reliabilities, and intercorrelations between the variables. All scales demonstrated satisfactory reliability (α and ω  =  0.71–0.94). Perceived stress positively correlated with PTSD (*r*  =  0.67, *p* < 0.001), PSC (*r*  =  0.54, *p* < 0.001), AAS (*r*  =  0.32, *p* < 0.001), and PC (*r*  =  0.52, *p* < 0.001). Except for the correlation between perceived stress and AAS, all coefficients represented a large effect. However, the AAS represented a medium effect. Therefore, high levels of perceived stress were associated with high levels of PTSD. By contrast, high levels of perceived stress were associated with low confidence in problem-solving ability, an avoidant problem-solving style, and the absence of PC during problem-solving, with higher scores on the PSI reflecting more negative appraisals of problem-solving ability.

**Table 1.** Intercorrelations, descriptive statistics, and reliabilities of the study variables.

Variable	1	2	3	4	5	6
1. Perceived stress	—					
2. PTSD	0.673**	—				
3. PSC	0.537**	0.487**	—			
4. AAS	0.323**	0.285**	0.595**	—		
5. PC	0.512**	0.507**	0.530**	0.504**	—	
6. Problem-solving appraisal	0.517**	0.475**	0.855**	0.893**	0.729**	—
Mean	23.89	38.46	29.90	48.43	19.52	97.85
SD	6.28	18.98	8.37	10.59	4.99	20.25
Alpha	0.85	0.94	0.85	0.79	0.71	0.89
Omega	0.86	0.94	0.85	0.81	0.71	0.89

Abbreviations: AAS, approach-avoidance style; PSC, problem-solving confidence; PTSD, posttraumatic stress disorder; PC, personal control; SD, standard deviation.

** *p* < 0.001.

Significant gender differences were found for some variables. For instance, women reported higher levels of perceived stress (*M*  =  24.25, *SD*  =  6.14) than those reported by men (*M*  =  22.26, *SD*  =  6.47, *t*  =  2.33, *p*  =  0.02). Additionally, women reported lower levels of PC (*M*  *=*  19.81, *SD*  =  4.86) than those reported by men (*M*  =  18.21, *SD*  =  5.20, *t*  =  2.37, *p*  =  0.02). Age negatively correlated with all variables (perceived stress: *r*  =  −0.32, *p* < 0.001; PTSD: *r*  =  −0.29, *p* < 0.001; PSC: *r*  =  −0.31, *p* < 0.001; AAS: *r*  =  −0.22, *p* < 0.001; and PC: *r*  =  −0.33, *p* < 0.001).

Table 2 presents the direct and indirect effects of perceived stress and the direct effects of the dimensions of problem-solving appraisal. All indirect effects, except for those of AAS, were significant:

- PSC partially mediated the relationship between perceived stress and PTSD (β  =  0.07, 95% CI [0.04, 0.40]).
- PC partially mediated the relationship between perceived stress and PTSD (β  =  0.09, 95% CI [0.11, 0.44]).
- AAS did not mediate the relationship between perceived stress and PTSD (β  =  −0.05, 95% CI [−0.17, 0.02]).

**Table 2.** Direct and indirect effects of perceived stress and problem-solving appraisal on PTSD.

**Effect**	***B***	**SE**	**95% CI**	***β***	***p***
*Direct effects*					
Stress → PTSD	0.72	0.06	[0.59, 0.84]	0.51	<0.001
Problem-solving appraisal → PTSD	0.15	0.05	[0.06, 0.24]	0.16	<0.001
PSC → PTSD	0.32	0.13	[0.07, 0.57]	0.14	0.013
AAS → PTSD	−0.13	0.09	[−0.30, 0.06]	−0.07	0.18
PC → PTSD	0.75	0.20	[0.36, 1.14]	0.20	<0.001
*Indirect effects*					
Stress → problem-solving appraisal → PTSD	0.23	0.15	[0.07, 0.38]	0.08	—
Stress → PSC → PTSD	0.21	0.09	[0.04, 0.40]	0.07	—
Stress → AAS → PTSD	−0.05	0.05	[−0.17, 0.02]	−0.02	—
Stress → PC → PTSD	0.27	0.08	[0.11, 0.44]	0.09	—

Abbreviations: AAS, approach-avoidance style; PTSD, posttraumatic stress disorder; SE, standard error; CI, confidence interval; PC, personal control; PSC, problem-solving confidence.

**Figure 1.**
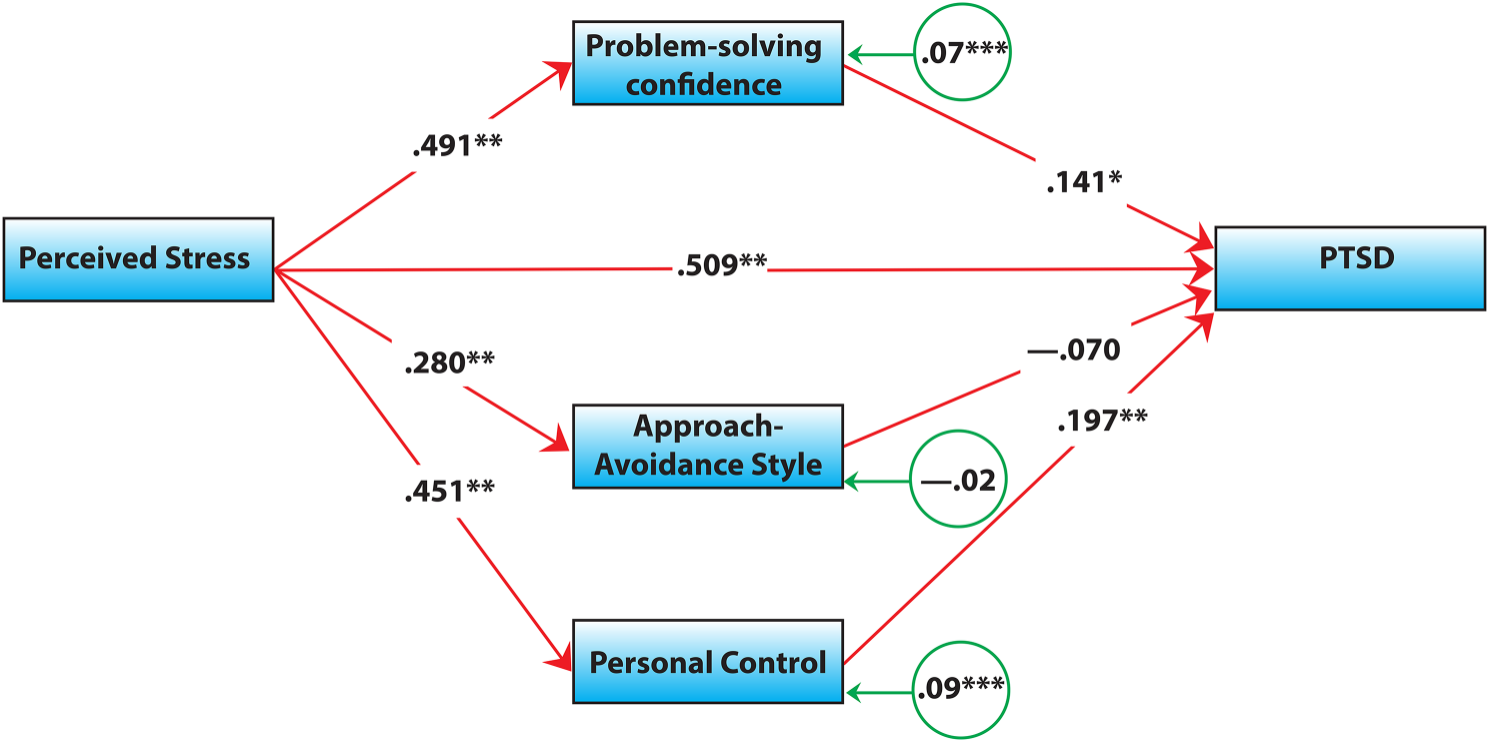
Visual representation of the mediating role of problem-solving appraisal.

*Note.* All coefficients are standardized. The circles indicate indirect effects. ** *p* < 0.001; * *p* < 0.05; *** evaluated with 95% confidence intervaals (CIs).

The direct effects of PSC (β  =  0.14, *p*  =  0.013) and PC (β  =  0.20, *p* < 0.001) on PTSD were significant. Figure 1 shows the results obtained using the PROCESS macro.

## Discussion

Dysfunctional cognitive appraisals have consistently been identified as strong predictors of psychological adjustment following exposure to trauma.^26^ However, only a few studies have investigated the role of adaptive cognitive appraisals in the relationship between stress and PTSD. In this study, we addressed this gap by investigating the role of self-appraised problem-solving ability in the relationship between stress and PTSD in a sample of university students.

First, we discovered that PSC partially mediated the relationship between stress and PTSD. Negative cognitive appraisals of a traumatic event (e.g., appraisals related to self-blame) and post-traumatic stress symptomology (e.g., appraising flashbacks as indicative of a permanent change in cognitive functioning), as well as the reactions of other people (e.g., appraising other people as untrustworthy), play a central role in the development and persistence of PTSD. Negative appraisals evoke adverse emotional reactions (e.g., fear, shame, guilt, and sadness) and foster avoidance behaviors that ultimately impede the processing of the traumatic event and maintain a negative view of the self.^26^ Hence, stronger beliefs and confidence in one's ability to solve a problem may generate an increased sense of agency in overcoming the impact of the trauma and mitigate the development of PTSD.

Previous research (e.g., Brown et al.^37^) on self-efficacy suggests that individuals tend to draw on past experiences when attempting to solve problems. A similar process may occur during problem-solving appraisal. Evoking memories of successful prior attempts at problem-solving may increase the level of confidence and evoke positive emotions. According to the broaden-and-build theory of positive emotion of Fredrickson,^38^ the experience of positive emotional states broadens momentary thought-action repertoires and weakens the grip of negative emotions on the individual, thus facilitating coping.

Second, we discovered that PC (i.e., the belief that one is in control of their behaviors and emotions) partially mediates the relationship between perceived stress and PTSD. The construct of PC is similar to that of perceived control, which has been extensively associated with physical and mental health outcomes.^39,40^ Perceived control plays a central role in PTSD.^8^ Specifically, control over a post-trauma recovery process is associated with improved adjustment.^41^ Theories of vulnerability (e.g., Gallagher et al.^42^) suggest that diminished perceived control is a key aspect of negative emotional experiences. Appraisals of PC may confer confidence in the ability to recover following exposure to stressors, thus generating a positive outlook and buffering against PTSD.

Finally, we discovered that the AAS (i.e., the general disposition to approach or avoid problem-solving tasks) does not mediate the relationship between perceived stress and PTSD. However, this finding is in contrast to existing research,^43,44^ which has consistently linked avoidant coping to psychological distress. Generally, the use of cognitive and behavioral avoidance strategies has been implicated in the persistence of PTSD, as these strategies hinder the processing of traumatic events in memory.^8^ One possible explanation for this finding is that PC may be the central mechanism influencing trauma-related outcomes. PC can enhance an individual's sense of agency, repertoire of coping strategies, and confidence in their ability to effectively cope with trauma-related cues, thereby ameliorating their PTSD symptoms. However, further longitudinal and qualitative studies are required to elucidate the associations among these variables. Qualitative studies on emotional regulation have reported that avoidance strategies (e.g., emotional suppression and distraction) can be effective in long-term adaptation. For example, Harnisch and Montgomery^45^ reported that personal silencing (i.e., deciding not to speak about the trauma to significant others) and suppression of emotions related to vulnerability formed part of effective coping repertoires among adults who were forcibly recruited as children into the Ugandan civil war. These strategies were found to reduce distress among loved ones and victims and prevent stigmatization and retaliatory responses once they returned to their communities.

Overall, our results have potential implications for clinical practice. Empirically supported treatments for PTSD focus on identifying and modifying idiosyncratic maladaptive appraisals of trauma and its sequelae.^8^ These interventions do not explicitly target appraisals related to problem-solving ability. However, feelings of helplessness and powerlessness represent core emotional responses to trauma and are elicited by appraisals of not being able to act or respond in an adaptive way (i.e., not being able to effectively solve a problem) and a loss of a sense of PC.^27^ Hence, a complementary focus of interventions can involve assessing problem-solving appraisals and developing and increasing adaptive or positive cognitions pertaining to PC and confidence in problem-solving ability. Therefore, with PC emerging as a salient factor, clinicians could potentially target specific appraisals that enhance perceptions of control. This could possibly form part of a broader process of rebuilding the individual's worldview following exposure to trauma. The PSI can also be used to monitor changes in appraisals of problem-solving ability, PC, and PSC to determine whether an intervention produces changes in symptomology. Although AAS did not emerge as a significant predictor of psychological distress in the present study, it is well established that cognitive and behavioral avoidance maintain symptoms of PTSD. Hence, one potential use of the PSI can, for example, be to determine if the individual is strongly avoidant (i.e., AAS) and how this could potentially maintain PTSD symptomology. Interventions to enhance their problem-solving appraisals can be monitored to determine if they lead to changes in PTSD symptomology by motivating the individual to approach rather than avoid the problem. This type of information can aid in tailoring interventions and thereby improving treatment outcomes.

This study has several methodological limitations, including its cross-sectional research design, which precluded the examination of the temporal associations between the study variables, and the use of self-report instruments, which are prone to selection and social desirability biases. Longitudinal research and the use of structured interviews to assess study variables may reduce bias and confirm the results. Additionally, the sample was recruited from a single site and predominantly consisted of women. Therefore, a larger and more representative sample is recommended to corroborate our findings.
